# *Rickettsia felis* in Fleas, France

**DOI:** 10.3201/eid1404.071103

**Published:** 2008-04

**Authors:** Jeremie Gilles, Frank Thomas Just, Cornelia Silaghi, Ingrid Pradel, Heidi Lengauer, Klaus Hellmann, Kurt Pfister

**Affiliations:** * Ludwig-Maximilians-University, Munich, Germany; † Klifovet AG, Munich, Germany; 1 Current affiliation: University of Kentucky, Lexington, Kentucky, USA.

**Keywords:** Rickettsia felis, France, polymerase chain reaction, Ctenocephalis felis, Ctenocephalis canis, Archaeopsylla erinacei, letter

**To the Editor:** *Rickettsia felis* belongs to the spotted fever group of rickettsia. The pathogenic role of this intracellular Proteobacteria in humans has been reported in patients from the United States (Texas) (*1*), Mexico (*2*), Germany (*3*), Brazil, and France (*4*). *R*. *felis* is widely distributed, is associated with blood-sucking arthropods, and has been isolated from fleas in several countries (*5*).

To obtain new information about the distribution of *R*. *felis* in France and potential vectors/ reservoirs of this emerging pathogen, 550 fleas were collected from 82 dogs and 91 cats in 7 widely distributed locations in France (Bordeaux, Toulouse, Cosnes-Cours sur Loire, Dijon, Moulins, Limoges, and Aix-en-Provence). Specimens were collected by combing, recorded, and stored at –20°C. Samples were shipped on dry ice to the entomologic laboratory of the Institute of Comparative Tropical Medicine and Parasitology in Munich, Germany, and species identification was performed by using light microscopy and following the determination key of Hopkins and Rothschild (*6*). Because infestation levels varied (1–150 fleas/animal), we randomly analyzed 1–8 fleas (mean 3.4) from each host animal.

We homogenized fleas individually in 80 μL of phosphate-buffered saline by using 5-mm steel beads in a RETSCH Tissue Lyser Mixer Mill 300 (QIAGEN, Hilden, Germany). A total of 100 μL of ATL buffer and 20 μL of proteinase K (QIAGEN) were added, and the homogenate was incubated at 56°C in a thermomixer (Eppendorf, Hamburg, Germany) until the tissues were lysed. DNA was extracted from each flea by using a QIAamp DNA Mini Kit (QIAGEN) according to the manufacturer’s instructions (tissue protocol) and stored at –20°C until used in a PCR.

PCR amplification of rickettsial DNA was performed by using previously described oligonucleotide primer pairs Rp CS.877p/Rp CS.1258n targeting the citrate synthase (*gltA*) gene and, for the positive samples, Rr 190.70p/Rr 190.602n targeting the outer membrane protein A (*ompA*) gene (*7*). Amplification was conducted in 50-μL volumes that contained 5 μL of DNA, 30 μL of distilled water, 10 μL of 5× Taq buffer (Roche, Mannheim, Germany), 3 μL of 25 mmol/L MgCl_2_ (Roche), 1 μL of 10 mmol/L deoxyncleotide triphosphates (Roche), 0.25 μL of each primer (100 μM), and 0.5 μL (5 U/mL) of Taq polymerase (Roche). Conditions for the *gltA* and *ompA* PCRs were as described by Bertolotti et al. (*8*). Negative and positive controls were included in all PCRs. All PCR products were separated by electrophoresis on 1.5% agarose gels at 100 V for 60 min and examined under UV light. For both genes, positive samples were purified by using the QIAquick PCR Purification Kit (QIAGEN) and sent for sequencing to the MWG Biotech Company (Martinried, Germany). Sequences were compared with those of previously characterized rickettsia in GenBank by using basic local alignment search tool (BLAST) (www.ncbi.nlm.nih.gov) analysis.

Five species of fleas were identified: *Ctenocephalides felis* (500, 224 from dogs and 276 from cats), *C*. *canis* (37 from dogs), *Pulex irritans* (11 from dogs), *Spilopsyllus cuniculi* (1 from a cat), and *Archaeopsylla erinacei* (1 from a cat). Five dogs had mixed populations of fleas; 3 of these had *P*. *irritans* and *C*. *felis*, and 2 had *C*. *felis* and *C*. *canis*. One cat had *P*. *irritans* and *C*. *felis*, and another cat had *S*. *cuniculi* and *C*. *felis*. A total of 52 (19%) of the 272 fleas from dogs and 44 (16%) of the 278 fleas from cats were positive for both the *gltA* and *ompA* genes. Positive samples were obtained from all locations. Prevalence ranged from 6% (Dijon) to 43% (Toulouse) for dogs and from 3% (Moulins) to 37% (Bordeaux) for cats (Table). Of 550 fleas, 96 were positive for both genes (*gltA* and *ompA*) and 3 of 5 species of fleas were infected: 10 with *C*. *canis*, 85 with *C*. *felis*, and 1 with *A*. *erinacei*. All sequences matched *gltA* and *ompA* genes from *R*. *felis* (similarity 99%–100%).

**Table:** Prevalence of *Rickettsia felis* in fleas from dogs and cats, France*

Locality	Animal	No. animals	No. fleas	Flea species	No. (%) *gltA*+ *ompA*+
Aix-en-Provence	Dog	6	20	*Ctenocephalis felis,*† *C. canis,*† *Pulex irritans*	6 (30)
Bordeaux	Dog	14	67	*C. felis,*† *C. canis, P. irritans*	8 (12)
	Cat	11	38	*C. felis†*	14 (37)
Cosnes-Cours sur Loire	Dog	15	44	*C. felis,*† *C. canis*	7 (16)
	Cat	17	50	*C. felis†*	3 (6)
Dijon	Dog	6	18	*C. felis,*† *C. canis*	1 (17)
	Cat	1	3	*C. felis†*	1 (33)
Limoges	Dog	15	45	*C. felis†*	7 (16)
	Cat	21	61	*C. felis,*† *Archaeopsylla erinacei*†	11 (18)
Moulins	Dog	12	36	*C. felis,*† *C. canis*	5 (14)
	Cat	22	65	*C. felis,*† *Spilopsyllus cuniculi*	2 (3)
Toulouse	Dog	14	42	*C. felis,*† *C. canis*†	18 (43)
	Cat	19	61	*C. felis†*	13 (21)

*gltA, citrate synthase A; *ompA*, outer membrane protein A. †Species positive for *gltA* and *ompA*.

Our investigation provides new information about distribution of *R*. *felis* and widespread flea infection with *R*. *felis* in France. A total of 88% of infected fleas were *C*. *felis*, but we found infected *C*. *canis* in Bordeaux and Toulouse and infected *A*. *erinacei* in Limoges. We report the presence in France of *R*. *felis* in *C*. *canis* and *A*. *erinacei* in France. *R*. *felis* in dog fleas in Uruguay and in hedgehog fleas in Algeria has been reported (*9*,*10*). Our findings indicate that these 2 flea species may be vectors of human *R*. *felis* rickettsiosis in France.
